# Epigenomic regulation of neural crest differentiation in human-induced pluripotent stem cells

**DOI:** 10.1016/j.isci.2026.115993

**Published:** 2026-05-22

**Authors:** Kyosuke Mukae, Maya Shindo, Tianyuan Shi, Ritsuko Onuki, Satoshi Yamashita, Naoko Hattori, Toshikazu Ushijima, Miki Ohira, Takehiko Kamijo

**Affiliations:** 1 Research Institute for Clinical Oncology, Saitama Cancer Center, Saitama, Japan; 2 Department of Graduate School of Science and Engineering, Saitama University, Saitama, Japan; 3 Department of Life Engineering, Faculty of Engineering, Maebashi Institute of Technology, Maebashi, Gunma, Japan; 4 Department of Epigenomics, Institute for Advanced Life Sciences, Hoshi University, Tokyo, Japan; 5 Laboratory of Integrative Metabolic Regulation, Institute for Molecular and Cellular Regulation, Gunma University, Maebashi, Gunma, Japan

**Keywords:** cellular neuroscience, stem cells research, omics

## Abstract

Neural crest cells (NCCs) drive vertebrate development through lineage specific differentiation into diverse tissues. Cranial (cNCCs) and trunk NCCs (tNCCs) exhibit distinct developmental trajectories that require coordinated epigenomic regulation. Aberrant NCC differentiation is associated with diseases including tumors. Epigenetic mechanisms such as histone modifications and DNA methylation are crucial for regulating NCC differentiation. Here, we used a human induced pluripotent stem cell model to compare differentiation of cNCCs and tNCCs and define lineage specific mechanisms. Integrated transcriptome and DNA methylome analyses revealed that DNA demethylation upstream of *MEF2C* in cNCCs and the *THRA* locus (a shared promoter region for *THRA1* and *THRA2* isoforms) in tNCCs was associated with increased expression of *MEF2C* and *THRA2*. MEF2C and THRA2 were associated with NCC markers. These findings define epigenomic features underlying lineage divergence and provide insight into NCC-related diseases such as leiomyosarcoma and neuroblastoma.

## Highlights

• DNA methylation regulates cranial and trunk neural crest differentiation
• MEF2C and THRA2 are linked to lineage-specific NCC gene expression
• Integrated transcriptome and methylome analyses reveal epigenomic programs
• Findings provide insight into NCC-related diseases including neuroblastoma

## Graphical abstract

**Figure:**
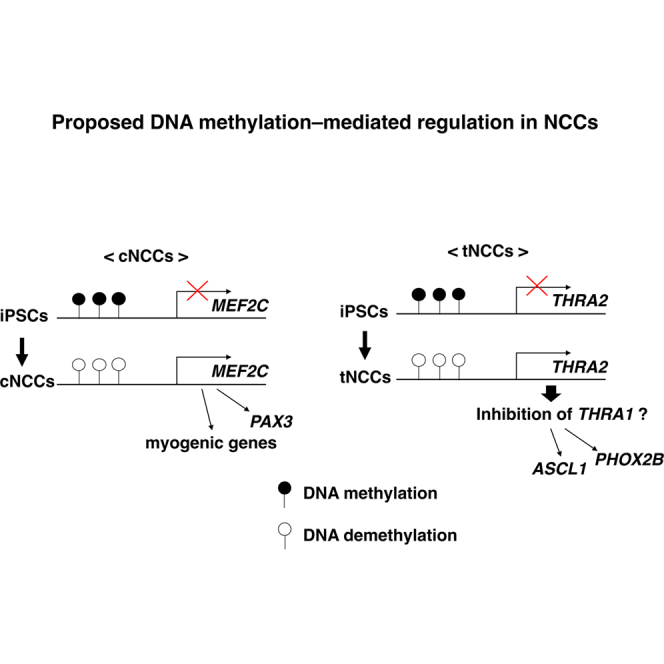

## Introduction

Neural crest cells (NCCs), derived from the neural tube during early embryogenesis, are a crucial cell population in vertebrate development. They primarily differentiate into cranial NCCs (cNCCs) or trunk NCCs (tNCCs). Subsequently, cNCCs differentiate into various tissues such as bone and cartilage. In contrast, tNCCs differentiate into sympathetic ganglia and melanocytes, contributing to the formation of diverse tissues and organs.^1^ Proper differentiation and fate specification of NCCs are essential for the normal development and survival of individuals. Aberrant differentiation of NCCs can lead to various diseases such as cardiac diseases and tumors.^2^^,^^3^

Jumonji domain-containing protein 2A regulates neural crest formation by modulating histone 3 lysine 9 trimethylation, which is an epigenetic factor that governs differentiation into NCCs.^1^ The transcription factors TFAP2A, NR2F1, and NR2F2 simultaneously bind to enhancer regions of NCC-specific genes, whereas the histone markers H3K27ac and H3K4me1 act synergistically to promote an active enhancer chromatin state.^4^ In addition to histone modifications, DNA methylation represents another fundamental epigenetic regulatory mechanism. During development, DNA methylation-mediated epigenetic repression serves as a ubiquitous mechanism that prevents the activation of alternative pathways during cell-type specification and lineage formation.^5^ Although somatic DNA methylation patterns are generally stably maintained, they undergo dynamic remodeling at specific developmental stages, such as during the preimplantation embryo.^6^^,^^7^ During mammalian development, DNA methylation is tightly regulated by conserved DNA methyltransferases (DNMTs). For example, *Dnmt1* knockout decreases the proliferation and subsequent differentiation of NCCs in mice.^8^ Similarly, DNA methyltransferase 3 beta (DNMT3B) is involved in NCC differentiation in humans, with its knockdown promoting neuronal and neural crest differentiation and upregulating the expression of NCC-specific genes.^9^ Notably, mutations in *DNMT3B* are associated with craniofacial abnormalities and neurological deficits related to neural crest development in humans.^10^ NCCs are initially formed within the central nervous system and subsequently migrate to form elements of the facial skeleton and peripheral nervous system. In neural tube progenitor cells, DNMT3B directly methylates the promoter region of the neural crest gene, *SOX10*, thereby inhibiting its transcription. Consequently, the absence of DNMT3B correlates in the overproduction of NCCs and premature neuronal differentiation in the peripheral nervous system.^10^ These findings highlight the importance of DNA methylation in regulating the capacity of neural tube cells to generate NCCs and the timing of peripheral neuronal differentiation.

Although DNA methylation analysis specific to NCC differentiation has not been reported, analysis of DNA methylation dynamics during *in vivo* neuronal differentiation has been conducted. This analysis revealed that the promoter regions of neurogenic transcription factors exhibit decreased levels of DNA methylation upon differentiation into the nervous system.^11^ This suggests that differentiation into NCCs, at least into tNCCs, is also potentially influenced by DNA methylation. Moreover, given the distinct differentiation potentials of cNCCs and tNCCs, their lineage specification may involve distinct DNA methylation patterns. During differentiation of embryonic stem (ES) cells, the promoter regions of pluripotency genes undergo DNA methylation, leading to the downregulation of gene expression. In contrast, the promoter regions of somatic cell genes remain unmethylated, maintaining gene expression.^12^^,^^13^ Moreover, *NANOG*, a key factor of induced pluripotent stem cells (iPSCs), exhibits low DNA methylation levels and an open chromatin configuration at its promoter region in ES cells.^14^ Notably, the differentiation of iPSCs into cNCCs is accompanied by a decrease in *NANOG* expression.^15^ However, the detailed regulatory mechanisms underlying this process remain elusive owing to the lack of DNA methylation and histone modification analyses.

Although the importance of histone modifications and DNA methylation in NCC differentiation has been reported,^1^ most of these studies have treated NCCs as a single population, and comparative analyses between cNCCs and tNCCs are lacking. Recently, protocols for inducing differentiation of human iPSCs into cNCCs and tNCCs have been established, each exhibiting a characteristic gene expression pattern.^15^^,^^16^ Although the use of human ES cells is restricted in certain countries owing to ethical concerns, human iPSCs are generally exempt from such limitations. Therefore, in the present study, we aimed to characterize DNA methylation patterns associated with NCC differentiation by comparing cranial and trunk NCCs derived from iPSCs, which we used as our model system. Because iPSCs may not fully recapitulate all developmental states observed in embryonic stem cells,^13^ potentially due to residual epigenetic memory,^13^ we employed three independent iPSC lines to minimize line-specific biases. We specifically focused on identifying somatic genes that undergo coordinated DNA demethylation and transcriptional upregulation during NCC differentiation.

## Results

### Preparation of cranial and trunk NCCs

**Figure 1:**
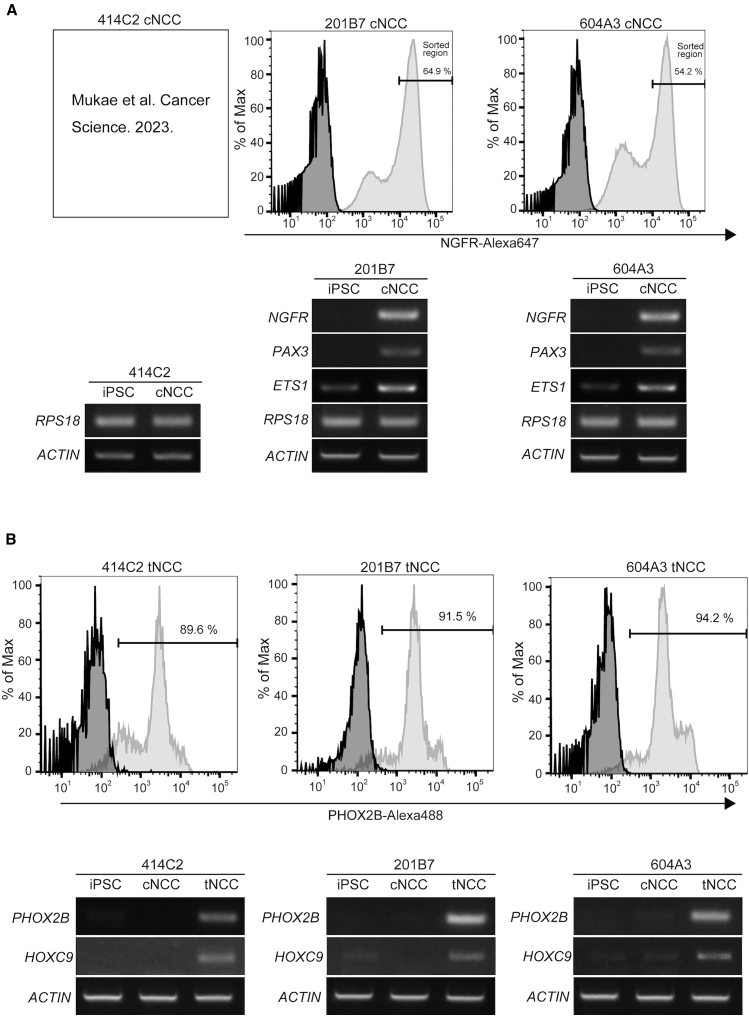
Induction of cNCCs and tNCCs from iPSCs (A) Induction of cNCCs. Human iPSC lines 201B7 and 604A3 were differentiated into cNCCs, stained with an Alexa Fluor 647 mouse anti-human CD271 (NGFR) antibody, and analyzed using FACS (black line: control antibody, gray line: CD271 antibody). NGFR-positive cells (>10^4^ fluorescence intensity) were sorted, and their proportion is shown. Semiquantitative RT-PCR was performed to assess the expression of neural crest markers (*NGFR*, *PAX3*, and *ETS1*) in iPSCs and cNCCs derived from 201B7 to 604A3. *ACTIN* and *RPS18* were used as a loading control. Semiquantitative RT-PCR data for *NGFR*, *PAX3*, and *ETS1* in 414C2 were reported previously.^17^ (B) Induction of tNCCs. Human iPSC lines 414C2, 201B7, and 604A3 were differentiated into tNCC, stained with anti-PHOX2B antibody, and analyzed using FACS (black line: control antibody, gray line: PHOX2B antibody). The proportion of PHOX2B-expressing cells is shown. Semiquantitative RT-PCR was performed to evaluate the expression of neural crest markers (*PHOX2B* and *HOXC9*) in iPSCs, cNCCs, and tNCCs. *ACTIN* was used as a loading control. *ACTIN* was used as a reference gene for tNCCs, while both *ACTIN* and *RPS18* were used for cNCCs due to potential cytoskeletal remodeling during cNCC differentiation. Abbreviations: cNCCs, cranial neural crest cells; tNCCs, trunk neural crest cells; iPSCs, induced pluripotent stem cells; FACS, fluorescence-activated cell sorting; RT-PCR, reverse transcription polymerase chain reaction; NGFR, nerve growth factor receptor; PAX3, paired box 3 ETS1, ETS proto-oncogene 1; ACTIN, beta-actin; RPS18, ribosomal protein S18; PHOX2B, paired-like homeobox 2 b; HOXC9, homeobox C9.

To define cNCC and tNCC populations derived from human iPSCs, we performed flow cytometric and molecular analyses using three independent iPSC lines (414C2, 201B7, and 604A3) (Figure S1). For cNCC identification, NGFR-positive populations were quantified using flow cytometry. Representative histograms demonstrated clear separation between isotype controls and NGFR-stained samples. The proportion of NGFR-positive cNCCs was 64.9% in 201B7 and 54.2% in 604A3 cells (Figure 1A). The characteristics of 414C2-derived cNCCs have been described previously,^17^ and comparable NGFR-positive populations were obtained in the present study. Sorted NGFR-positive cells exhibited enriched expression of canonical cNCC markers, including *NGFR*, *PAX3*, and *ETS1*, as confirmed using RT-PCR analysis. For tNCCs identification, PHOX2B-positive populations were analyzed using flow cytometry. The proportion of PHOX2B-positive cells reached 89.6%, 91.5%, and 94.2% in 414C2, 201B7, and 604A3 lines, respectively (Figure 1B). RT-PCR analysis confirmed expression of tNCC-associated markers, including *PHOX2B* and *HOXC9*, in tNCC populations compared with iPSCs and cNCCs. Based on these molecular characterizations, NGFR-positive cells and PHOX2B-positive cells were defined as cNCC and tNCC populations, respectively, and were used for subsequent experiments.

### Cranial and trunk NCCs exhibit specific gene expression patterns

**Figure 2:**
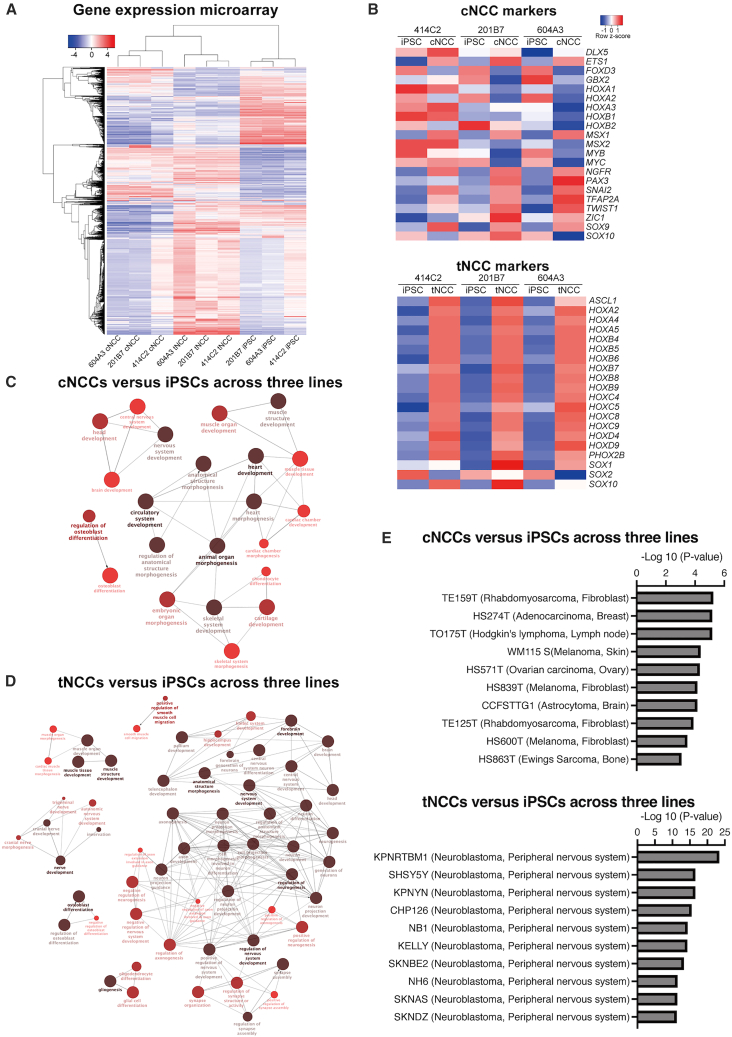
Gene expression analysis in iPSCs, cNCCs, and tNCCs (A) Heatmap of the gene expression profiles in iPSCs, cNCCs, and tNCCs. Data represent the mean of three technical replicates for each of three independent iPSC, cNCC, and tNCC lines. The heatmap was generated using 10,000 highly variable probes identified using iDEP (version 9.6). (B) Heatmap showing the mean expression levels of representative cNCC markers in iPSCs and cNCCs (upper panel), and tNCC markers in iPSCs and tNCCs (lower panel), based on gene expression microarray data. Data from all three independent iPSC lines are shown. Marker genes include established cNCC and tNCC markers and *HOX* genes, enabling comparison of lineage identity and differentiation stage among the cell populations. (C) Network analysis of genes showing a > 2-fold increase in cNCCs relative to iPSCs, consistently across all three iPSC-cNCC pairs. The analysis was performed using Cytoscape ClueGO, based on the genes listed in Table S1 (*p* < 0.001). (D) Network analysis of genes showing a >2-fold increase in tNCCs relative to iPSCs, consistently across all three iPSC-tNCC pairs. The analysis was performed using Cytoscape ClueGO, based on the genes listed in Table S2. (E) Enrichment analysis using Enrichr for cancer-related gene sets, based on common genes that exhibited a >2-fold increase in expression in cNCCs or tNCCs relative to iPSCs, consistently across all three iPSC-NCC pairs (*p* < 0.05). Abbreviations: iPSCs: induced pluripotent stem cells; cNCCs, cranial neural crest cells; tNCCs, trunk neural crest cells.

The 414C2, 201B7, and 604A3 iPSCs, derived from healthy individuals, were differentiated into cNCCs or tNCCs. Clustering analysis of 10,000 genes revealed distinct clusters corresponding to iPSC, cNCC, and tNCC, each characterized by specific sets of highly expressed genes (Figure 2A). Comparison of representative cNCC markers between iPSCs and cNCCs showed that not all markers were uniformly upregulated in cNCCs. This finding is consistent with previous reports demonstrating heterogeneity in cNCC marker expression during differentiation (Figure 2B upper panel).^15^ In contrast, analysis of tNCC markers revealed consistent upregulation of nearly all examined markers in tNCCs relative to iPSCs (Figure 2B lower panel).^18^ Gene ontology (GO) analysis of genes exhibiting more than 2-fold increased expression in cNCCs compared with iPSCs consistently across all three iPSC-cNCC pairs, identified significantly enriched pathways (*p* < 0.01) associated with cartilage, nerve, and muscle development (Table S1). Functional network analysis using Cytoscape ClueGo (*p* < 0.001) further demonstrated that musculoskeletal tissues formed prominent clusters in cNCCs (Figure 2C). Similarly, GO analysis of tNCCs revealed enrichment of nervous system-related pathways (Table S2). Network analysis identified a large cluster centered on nervous system functions (Figure 2D). Moreover, gene expression patterns exhibited a potential for differentiation toward neural lineages in both cNCCs and tNCCs. In terms of gene expression levels, cNCCs exhibited gene signatures similar to those of fibroblastic lineage tumors, such melanomas of cNCC origin, suggesting their potential for mesenchymal differentiation (Figure 2E upper panel; Table S3). In contrast, tNCCs displayed profiles resembling those of neuroblastoma, suggesting their origin from the tNCC and their potential to differentiate into the sympathoadrenal lineage (Figure 2E lower panel; Table S4). Moreover, transcription factor enrichment analysis identified 1,076 and 823 significant transcription factors in cNCCs and tNCCs, respectively (Tables S5 and S6). Among these, 759 transcription factors were commonly detected in both cNCCs and tNCCs, while 317 were specific to cNCCs and 64 were specific to tNCCs. The tNCC-specific set included *NEUROD1*, *NEUROD2*, and *NEUROD6*, transcription factors involved in neural development, providing evidence that differentiation into tNCCs are influenced by neuronal lineage-associated factors.

### DNA methylation analysis for NCC differentiation

**Figure 3:**
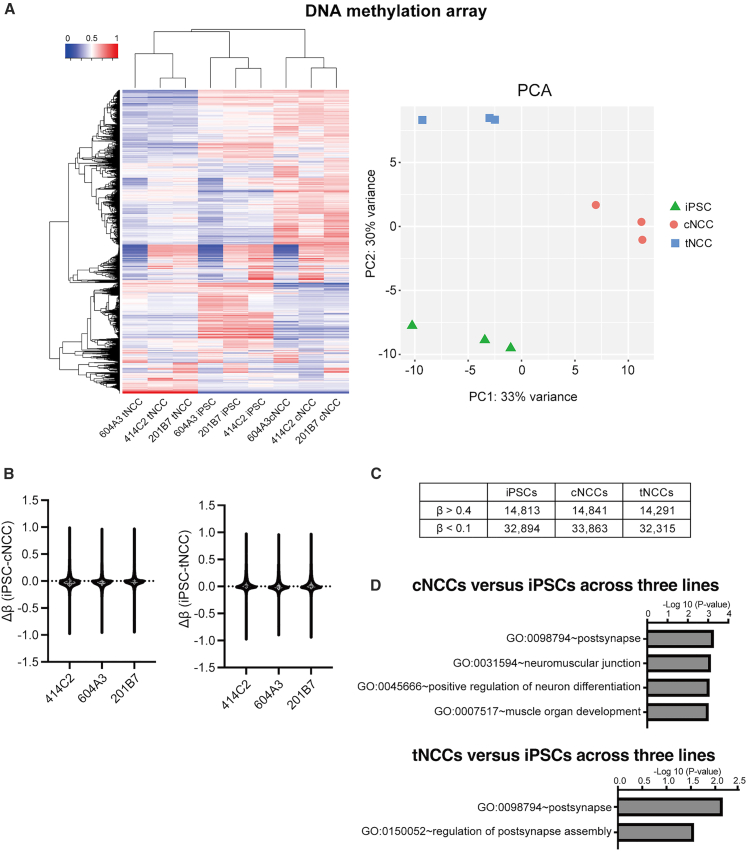
DNA methylation analysis in iPSCs, cNCCs, and tNCCs (A) Heatmap and principal component analysis of iPSCs, cNCCs, and tNCCs analyzed using the Infinium HumanMethylationEPIC microarray (*n* = 1 per line). DNA methylation levels are shown as β values ranging from 0 (green) to 1 (red). A total of 57,248 probes were selected based on CpG islands defined by the UCSC Genome Browser, located within 200 bp upstream of the transcription start site and within the 5′UTR and first exon. The heatmap was generated using the 10,000 most highly variable probes identified with iDEP (version 9.6). (B) Violin plots show the distribution of Δβ values (iPSC-cNCC and iPSC-tNCC) for three independent iPSC lines (414C2, 604A3, and 201B7). iPSCs were used as a common reference to visualize methylation changes associated with differentiation toward cNCCs and tNCCs. Each violin represents Δβ values calculated from paired biological samples (*n* = 1 per line), without averaging across lines. Positive and negative Δβ values indicate higher methylation levels in iPSCs or NCCs, respectively. (C) Number of DNA-methylated (β > 0.4) and demethylated (β < 0.1) genes mapped from CpG sites in three iPSCs, cNCCs, or tNCCs. (D) Promoter regions consistently demethylated in cNCCs or tNCCs relative to iPSCs across all three matched iPSC-NCC pairs were identified, and the associated genes were analyzed using DAVID. Abbreviations: iPSCs: induced pluripotent stem cells; cNCCs, cranial neural crest cells; tNCCs, trunk neural crest cells; CpG, cytosine-phosphate-guanine dinucleotide; UCSC, University of California, Santa Cruz; 5′UTR, 5′ UTR; Δβ, difference in DNA methylation β values; DAVID, database for annotation, visualization, and integrated discovery.

We conducted DNA methylation profiling of iPSCs, cNCCs, and tNCCs derived from the 414C2, 201B7, and 604A3 cell lines. Clustering analysis revealed distinct DNA methylation patterns for iPSCs, cNCCs, and tNCCs (Figure 3A; left). The DNA methylation pattern of 604A3 cells differed from those of 414C2 and 201B7 cells, suggesting the line-specific epigenetic characteristics of 604A3 cells. Principal-component analysis (PCA) further demonstrated a clear separation of iPSCs from both cNCCs and tNCCs (Figure 3A, right). To quantify the magnitude of methylation changes, we calculated effect sizes as Δβ values for each line (Δβ [iPSC-cNCC] and Δβ [iPSC-tNCC]). Violin plot analysis showed highly comparable Δβ distributions across all three independent cell lines, with no evident line-specific bias, indicating consistent differentiation-associated methylation shifts (Figure 3B). To further characterize these DNA methylation changes, we identified genes with highly methylated (β > 0.4) and demethylated (β < 0.1) promoter regions in iPSCs, cNCCs, and tNCCs across all three cell lines (Figure 3C). To validate the biological relevance of this classification, we examined the methylation status of well-characterized pluripotency-associated transcription factors. The core pluripotency transcription factors *POU5F1*, *SOX2*, *KLF4*, and *MYC* are known to be unmethylated in embryonic stem (ES) cells.^19^ Consistent with this, the promoter-proximal regions upstream of the transcription start sites (TSSs) of *SOX2*, *KLF4*, and *MYC* exhibited low methylation levels in iPSCs, which were maintained following differentiation into cNCCs and tNCCs (Table S7). No CpG islands were identified within the promoter region of *POU5F1*. Furthermore, functional annotation analysis using database for annotation, visualization, and integrated discovery (DAVID) revealed that demethylated genes in cNCCs were enriched for pathways related to neural and muscular functions, whereas those in tNCCs were predominantly associated with neural-related pathways (Figure 3D).

**Figure 4:**
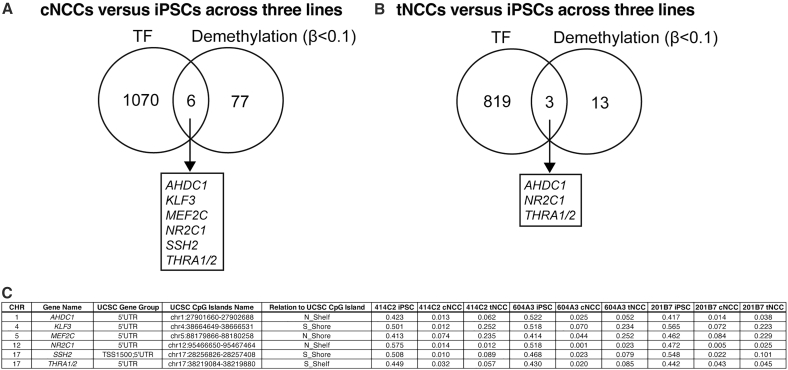
Identification of candidate transcription factors associated with DNA demethylation in cNCCs and tNCCs (A) Common genes identified by comparing the 1,076 transcription factor (TF) genes listed in Table S5 with the 83 TF genes that underwent DNA demethylation (β < 0.1) upstream of the first exon during differentiation into cNCCs. The six overlapping genes are presented at the bottom of the Venn diagram. (B) Common genes identified by comparing the 823 genes listed in Table S6 with the 16 genes that underwent DNA demethylation upstream of the first exon during differentiation into tNCCs. The four overlapping genes are presented at the bottom of the Venn diagram. (C) The table presents the TF genes along with their chromosomal positions, UCSC gene group classifications, UCSC CpG Islands, their relationship to UCSC CpG Islands, and β values. Abbreviations: iPSCs: induced pluripotent stem cells; cNCCs, cranial neural crest cells; tNCCs, trunk neural crest cells; CpG, cytosine-phosphate-guanine dinucleotide; UCSC, University of California, Santa Cruz, TF: transcription factor.

We next extracted genes that transitioned from a highly methylated state in iPSCs (β > 0.4) to a demethylated state in either cNCCs or tNCCs (β < 0.1) with consistent directional changes observed across all three matched iPSC-derived lines. GO analysis of these genes using DAVID revealed significant enrichment of neural-related terms in both cNCCs and tNCCs (Tables S8, S9, S10, and S11). In cNCCs, enriched categories included postsynapse, neuromuscular junction, positive regulation of neuron differentiation, and muscle organ development, whereas those in tNCCs included postsynapse and regulation of postsynapse assembly. These findings indicate that the regulation of DNA methylation plays a key role in guiding differentiation toward neural lineages (Tables S9 and S11). Finally, we examined the potentially active transcription factors that were associated with DNA-demethylated regions upstream of the first exon (TSS200, 5′UTR, and first exon), as listed in Tables S5 and S6. In cNCCs, demethylated regions were identified in *AHDC1*, *KLF3*, *MEF2C*, *NR2C1*, *SSH2*, and *THRA1*/*2* (Figure 4A). In tNCCs, demethylation was observed in *AHDC1*, *NR2C1*, and *THRA1*/*2* (Figure 4B). Among these, *AHDC1*, *NR2C1*, and *THRA1*/*2* were commonly demethylated in both cNCCs and tNCCs (Figure 4C). These findings suggest that multiple transcription factors are potentially modulated by DNA methylation during NCC differentiation.

### Integrated analysis of gene expression and DNA methylation

**Figure 5:**
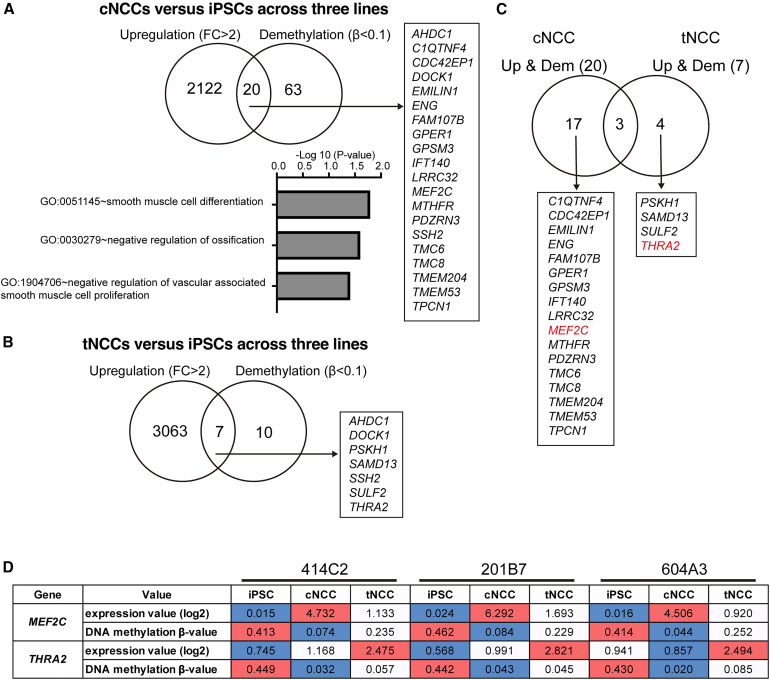
Integrated analysis of DNA demethylation and upregulated genes (A and B) Genes commonly upregulated (>2-fold expression) and DNA-demethylated (β < 0.1 at promoter regions) in cNCCs or tNCCs relative to iPSCs, consistently across all three iPSC-NCC pairs. A total of 20 genes (cNCCs) and 7 genes (tNCCs) met these criteria and were subjected to functional annotation analysis using DAVID to identify enriched developmental pathways (*p* < 0.05). (C) Genes specific to either cNCCs (17 genes) or tNCCs (4 genes) were extracted from the integrated dataset. Genes shown in red indicate transcription factors. (D) Expression levels of *MEF2C* and *THRA2* and β values in 5′UTR are presented in the table. Red indicates high expression or high DNA methylation levels, and blue indicates low expression or low methylation levels. Abbreviations: iPSCs: induced pluripotent stem cells; cNCCs, cranial neural crest cells; tNCCs, trunk neural crest cells; MEF2C, myocyte enhancer factor 2C; THRA, thyroid hormone receptor alpha; 5′UTR, 5′ UTR.

To investigate the functional relationship between DNA methylation and gene expression in cNCCs and tNCCs, we performed Pearson’s correlation analysis between the β values of differentially methylated CpG sites identified during cNCC and tNCC differentiation and expression levels of the corresponding differentially expressed genes (DEGs) (Table S12). Fifty-five upregulated genes exhibited a significant negative correlation with DNA methylation levels (r < 0, *p* < 0.05), indicating that demethylation may contribute to their transcriptional activation. Conversely, 31 downregulated genes exhibited a significant positive correlation (r > 0, *p* < 0.05), suggesting that increased methylation is associated with transcriptional repression. These findings support a regulatory role for DNA methylation in shaping the gene expression dynamics during cNCC and tNCC differentiation. Next, we focused on genes showing over a 2-fold upregulation in cNCCs or tNCCs compared with iPSCs, as well as those that were demethylated in cNCCs or tNCCs compared with iPSCs. The analysis identified 20 genes in cNCCs and 7 in tNCCs, which were subsequently subjected to pathway enrichment analysis (Figures 5A and 5B; Tables S13 and S14). In cNCCs, the significantly enriched pathways were associated with the nervous system and smooth muscle tissue, whereas in tNCCs, no differentiation-related pathway was identified (Figures 5A and 5B). Analysis of the transcription factors of the genes specific to cNCC (*n* = 20) and tNCC (*n* = 7) identified *MEF2C*, a transcriptional effector and target of endothelin signaling,^20^ and *THRA2*, which is involved in neural development (*THRA* variant 2),^21^ as the lineage-specific transcription factors, respectively (Figure 5C). Based on the integrated methylation and expression data, we hypothesized that these transcription factors play distinct roles in the differentiation of cNCCs and tNCCs. Therefore, these transcription factors were selected for further analysis as candidate regulators of NCC differentiation. Comparison of microarray gene expression data and β values from DNA methylation data across iPSCs, cNCCs, and tNCCs revealed upregulation of*MEF2C* in cNCCs and *THRA2* in tNCCs, which coincided with the demethylation of their respective promoter regions (Figure 5D).

### Downstream analysis of MEF2C for cNCCs and THRA2 for tNCCs

**Figure 6:**
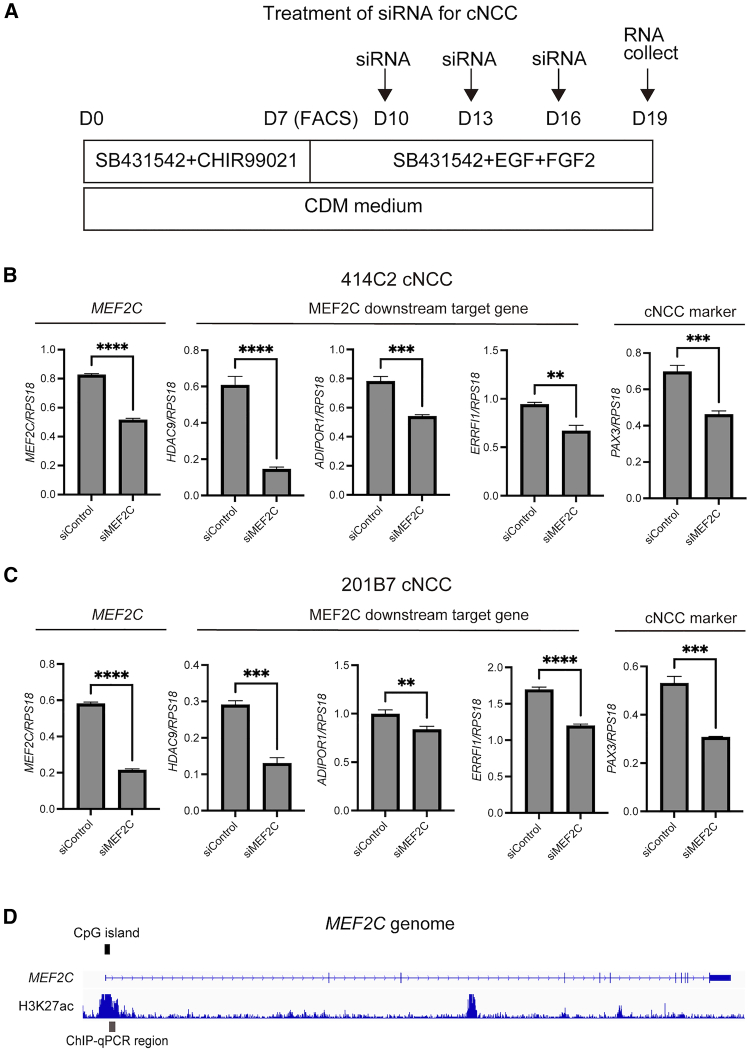
Change in downstream gene expression in response to *MEF2C* inhibition (A) siRNA treatment was initiated in cNCCs 3 days after FACS. (B and C) Gene expression levels were normalized to that of *RPS18* as a reference gene. Data are presented as mean ± SD from three technical replicates. Statistical significance was determined using Student’s *t* test comparing the experimental groups with the siControl group in 414C2 (B) and 201B7 (C) cNCCs. Differences were considered statistically significant at ∗∗*p* < 0.01, ∗∗∗*p* < 0.001, ∗∗∗∗*p* < 0.0001. (D) Genomic organization of the *MEF2C* locus showing gene structure, CpG island distribution, and H3K27ac enrichment. The upper track represents the *MEF2C* gene model, and the lower track shows H3K27ac ChIP-seq signal derived from muscle cells (GSM733639). Black bars indicate CpG islands. Gray bars represent the regions analyzed by ChIP-qPCR in this study. Abbreviations: cNCCs, cranial neural crest cells; siRNA, small interfering RNA; FACS, fluorescence-activated cell sorting; SD, standard deviation; siControl, control small interfering RNA; MEF2C, myocyte enhancer factor 2C; ChIP-seq, chromatin immunoprecipitation sequencing; ChIP-qPCR, chromatin immunoprecipitation quantitative polymerase chain reaction.

siRNA treatment was performed three times starting three days after cNCC induction to investigate the functional roles of *MEF2C* in cNCCs (Figure 6A). Next, we analyzed the expression of the downstream genes of MEF2C, as well as NCC markers. To assess MEF2C-mediated regulatory effects in cNCCs, we examined *ADIPOR1*, *ERRFI1*, and *HDAC9*, which are reported downstream targets involved in myogenic regulation.^22^^,^^23^^,^^24^ In both 414C2 and 201B7 cNCCs, siRNA-mediated knockdown of *MEF2C* (siMEF2C) significantly reduced the expression of *HDAC9*, *ADIPOR1*, and *ERRFI1*, which are downstream target genes of MEF2C (Figures 6B and 6C; Figure S2). We also analyzed *PAX3*, a marker of NCCs and myogenesis,^25^ and observed significant downregulation following *MEF2C* knockdown (Figures 6B and 6C). In addition, we examined the levels of H3K27ac, a marker of active chromatin, to assess epigenetic changes during cNCC differentiation (Figure 6D). No significant differences in H3K27ac enrichment were observed between iPSCs and cNCCs at the analyzed regions (Figure S3).

**Figure 7:**
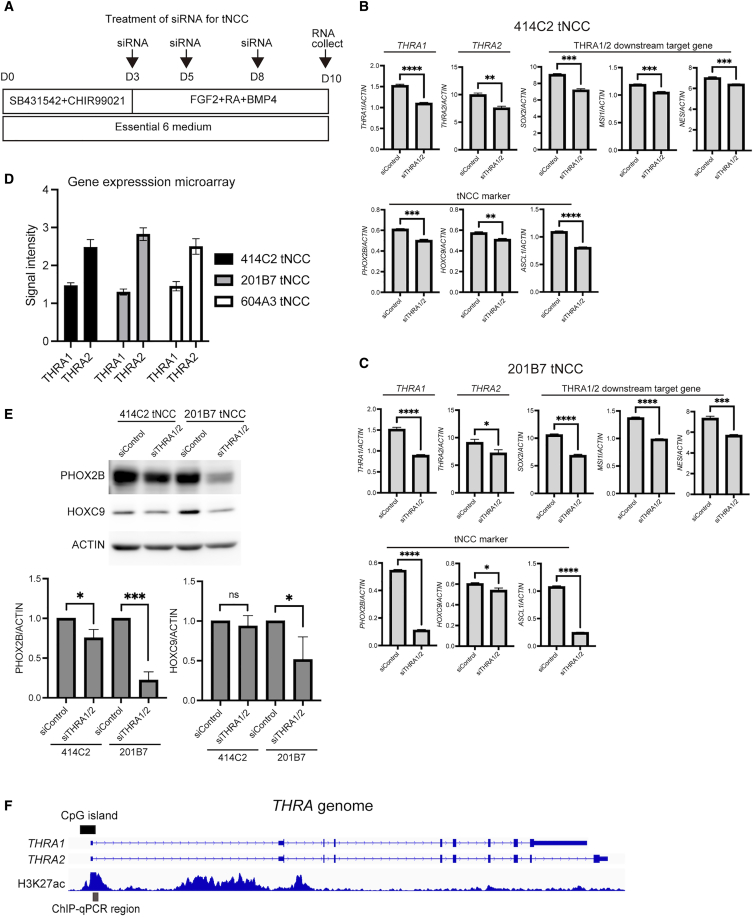
Changes in downstream gene expression following *THRA1*/*2* inhibition (A) In tNCCs, siRNA treatment was initiated immediately after induction of differentiation and repeated every 2 to 3 days until completion of differentiation. (B and C) Gene expression levels were normalized to that of *ACTIN* as the reference gene. Data are presented as mean ± SD from three technical replicates. Statistical significance was determined using Student’s *t* test by comparing each experimental group with the siControl group in 414C2 (B) and 201B7 (C) tNCCs. (D) Gene expression levels of *THRA1* and *THRA2* in tNCCs derived from 414C2, 201B7, and 604A3 extracted from the microarray data and presented as signal intensity. (E) Differences in the expression of HOXC9 and PHOX2B between the siControl- and siTHRA1/2-transfected 414C2 and 201B7 tNCCs (upper panel). ACTIN was used as the loading control (expression set to 1). Band intensities were quantified using ImageJ (lower panel). Data are presented as mean ± SD from three technical replicates. Differences were considered statistically significant at ∗*p* < 0.05, ∗∗*p* < 0.01, ∗∗∗*p* < 0.001, ∗∗∗∗*p* < 0.0001. (F) Genomic organization of the *THRA* locus showing the transcript structures of *THRA1* and *THRA2*, CpG island distribution, and H3K27ac enrichment. The upper tracks depict the gene models of *THRA1* and *THRA2*, and the lower track shows the H3K27ac ChIP-seq signal derived from BT16 cells (GSM5311619). Black bars indicate CpG islands. Gray bars represent the regions analyzed by ChIP-qPCR in this study. Abbreviations: tNCCs, trunk neural crest cells; siRNA, small interfering RNA; SD, standard deviation; siControl, control small interfering RNA; THRA, thyroid hormone receptor alpha; PHOX2B, paired-like homeobox 2 b; HOXC9, homeobox C9, ChIP-seq, chromatin immunoprecipitation sequencing; ChIP-qPCR, chromatin immunoprecipitation quantitative polymerase chain reaction.

For tNCCs, siRNA transfection was initiated at the onset of differentiation and performed three times to investigate the functional roles of *THRA2* in tNCCs (Figure 7A). Because of the high sequence similarity between *THRA1* and *THRA2*, isoform-specific targeting of THRA2 was technically challenging. Therefore, we simultaneously knocked down both isoforms. As THRA1 and THRA2 are functionally antagonistic, this approach does not allow discrimination of isoform-specific effects; thus, the observed phenotypes may reflect combined loss of both isoforms or an altered balance between them. THRA1 suppresses downstream genes such as *SOX2*, *NES*, and *MSI1*,^26^ whereas THRA2 counteracts these effects by inhibiting THRA1-mediated transcription. Inhibition of *THRA1*/*2* by siTHRA1/2 significantly suppressed *THRA1*/*2* and the downstream genes compared with siControl in both 414C2 and 201B7 tNCCs (Figures 7B and 7C). In addition, the gene expression microarray results indicated that *THRA2* is more abundant than *THRA1* in tNCCs (Figure 7D), suggesting that *THRA1*/*2* suppression may primarily affect downstream gene expression through *THRA2* inhibition. However, because *THRA1* and *THRA2* were simultaneously suppressed, the observed transcriptional and phenotypic changes may reflect the combined loss of both isoforms or an altered balance between them, rather than the loss of *THRA2* alone. Therefore, although THRA2 is more abundantly expressed in tNCCs, the relative contribution of each isoform cannot be definitively distinguished in the present experimental setting. Furthermore, *THRA1*/*2* inhibition significantly downregulated the tNCC markers *HOXC9*, *PHOX2B*, and *ASCL1* (Figures 7B and 7C). Similarly, PHOX2B expression levels were significantly reduced by siTHRA1/2 (Figure 7E). HOXC9 expression levels were significantly reduced in *THRA1*/*2* in 201B7 tNCCs but not in 414C2 tNCCs. This cell line–dependent difference suggests that the regulatory effect of THRA1/2 on HOXC9 may not be uniform across tNCC lines. Therefore, while *THRA1/2* suppression is associated with reduced PHOX2B expression in both lines, its effect on HOXC9 appears to be context-dependent. These findings suggest that THRA1/2 activity is associated with PHOX2B expression and may contribute to tNCC differentiation.

## Discussion

### Regulation of DNA methylation in NCCs

Loss of DNMT3B has been reported to result in the overproduction of NCCs and premature neuronal differentiation in the peripheral nervous system, underscoring the importance of DNA methylation in regulating the ability of neural tube cells to generate NCCs and the timing of peripheral neuronal differentiation.^10^ In the present study, we compared genes that were demethylated and exhibited increased expression in cNCCs or tNCCs relative to iPSCs. To the best of our knowledge, the regulation of gene expression via DNA methylation during NCC differentiation has not been previously reported. Our findings reveal a previously unrecognized role for DNA methylation in controlling differentiation of both cNCCs and tNCCs, suggesting that epigenetic regulation contributes to NCC lineage specification. In addition, we identified several other genes exhibiting both DNA demethylation and upregulation, providing further evidence for the critical role of DNA methylation regulation in NCC differentiation. Among the identified genes, *CDC42EP1*, *MEF2C*, and *DOCK1* in cNCCs and *DOCK1*, *SULF2*, and *THRA2* in tNCCs have been reported to be associated with NCC differentiation.^20^^,^^21^^,^^27^^,^^28^^,^^29^ Although we did not perform a time course analysis, our observation of increased gene expression during differentiation, along with DNA demethylation detected in iPSCs, suggests a strong association between demethylation and the transcriptional activation of these genes. Furthermore, differentially methylated region (DMR) analysis of putative enhancer regions between tNCCs and cNCCs identified genes with marked differences in methylation levels (Table S15). Specifically, *COL5A1*, *TFAP2B*, and *SPSB4* exhibited consistently lower Δβ values (<−0.3) in tNCCs compared with cNCCs, whereas *CUBN*, *TGFBI*, and *MMP9* showed consistently higher Δβ values (>0.3) in tNCCs compared with cNCCs. These genes have previously been implicated in NCC differentiation.^30^^,^^31^^,^^32^^,^^33^^,^^34^^,^^35^ Collectively, these findings support the hypothesis that DNA methylation plays a key regulatory role in NCC differentiation.

### Beyond H3K27ac: Multilayered regulation of MEF2C and THRA expression

We next examined whether changes in histone activation marks accompany transcriptional regulation of *MEF2C* and *THRA* during NCC differentiation. Although H3K27ac is widely used as a marker of active promoters and enhancers, ChIP-quantitative PCR analysis did not reveal a consistent increase in H3K27ac enrichment at the promoter-proximal regions of either gene during differentiation (Figure S3). These findings indicate that the changes in promoter-associated H3K27ac alone cannot fully explain the altered expression of *MEF2C* and *THRA*. One possibility is that transcriptional regulation is mediated through distal regulatory elements, such as enhancers, rather than promoter-proximal chromatin modifications. Alternatively, other epigenetic modifications—including additional histone modifications (H3K4me3, H3K9ac, or repressive marks such as H3K27me3), chromatin accessibility, transcription factor binding, or higher-order chromatin architecture—may contribute to the regulation of these genes. Together, these findings underscore the complexity of epigenetic regulation during NCC differentiation and suggest that multiple regulatory layers act in concert to control *MEF2C* and *THRA* expression.

### Single-cell perspectives on neural crest cell heterogeneity

A major limitation of the present study is the use of bulk transcriptomic and methylome analyses, which capture average signals across heterogeneous cell populations. Consistent with this limitation, GO analysis revealed enrichment of both neural and muscular pathways in cNCCs (Figures 2C and 3D), suggesting cellular heterogeneity. Accordingly, prominent signals observed in bulk analyses, such as *MEF2C* upregulation, may originate from specific subpopulations rather than being uniformly present across all cNCCs (Figure 2B). While this limitation does not undermine the observed associations, it warrants caution when interpreting lineage-specific conclusions. To further contextualize our findings, we analyzed publicly available single-cell RNA sequencing (RNA-seq) datasets of cNCCs and tNCCs from mouse and human. This exploratory analysis revealed preferential expression of *Mef2c* in mouse cNCCs and relatively higher expression of *THRA* in human tNCCs, consistent with our bulk transcriptome and methylome data (Figure S4). Together, these observations support lineage-associated regulatory differences while highlighting the heterogeneity within NCC populations. Future studies using single-cell multi-omics approaches are warranted to precisely map epigenetic and transcriptional signatures to distinct NCC subtypes.

### Effects of pharmacological or genetic inhibition on differentiation

We focused on *MEF2C* in cNCCs and *THRA2* in tNCCs as candidate transcription factors whose expression may be associated with changes in DNA demethylation status. In cNCCs, *MEF2C* knockdown led to a decrease in the expression of *MEF2C* downstream genes, including *HDAC9*, *ADIPOR1*, and *ERRFI1*, which are involved in myogenesis.^22^^,^^23^^,^^24^ Furthermore, *PAX3*, a marker of NCCs and myogenesis, was downregulated. These findings suggest that *MEF2C* is potentially regulated by DNA methylation and plays an indispensable role in maintaining the gene expression program of cNCCs. Furthermore, MEF2C has been implicated in myoblast differentiation,^36^ which was also supported by our findings. In contrast, we noted no decrease in PAX3 levels upon *MEF2C* inhibition, suggesting the existence of additional regulatory mechanisms for PAX3 expression (data not shown).

THRA1 is a nuclear receptor that is activated upon binding to thyroid hormone (T3) and regulates transcription by binding to thyroid hormone response elements (TREs). *THRA1* is expressed in various organs, including the heart, lungs, and kidneys. In contrast, *THRA2*, despite belonging to the same thyroid hormone receptor family, cannot bind to T3. Therefore, unlike THRA1, THRA2 does not mediate T3-dependent transcriptional regulation; instead, it competitively inhibits THRA1 by binding to TREs. Moreover, *THRA2* is highly expressed in neural tissues.^37^ In mice, *Thra1* suppression without altering *Thra2* expression results in the upregulation of *Sox2*, *Nes*, and *Msi1*, indicating that *Thra1* is a negative regulator of these genes.^26^ In contrast, in the present study, the simultaneous knockdown of both *THRA1* and *THRA2* in tNCCs led to a decrease in the expression of *SOX2*, *NES*, and *MSI1*. Because microarray data indicated higher expression of *THRA2* than *THRA1* in tNCCs, siTHRA1/2 likely preferentially targeted *THRA2* owing to its relatively high cellular abundance. Consequently, *THRA2* inhibition may have impaired the competitive effect of *THRA1*, resulting in the downregulation of *SOX2*, *NES*, and *MSI1*. Furthermore, *THRA2* knockdown suppressed the expression of all NCC markers, including *HOXC9*, *PHOX2B*, and *ASCL1*. Although *THRA2* expression was similarly reduced in tNCCs following siTHRA1/2 treatment, the expression levels of tNCC markers differed between the two cell types (414C2 and 201B7). This discrepancy may reflect intrinsic differences between the cell lines; however, the precise mechanisms remain unclear. Overall, these findings suggest that *THRA2* expression is associated with maintenance of tNCC identity and may be influenced by DNA methylation. However, selective inhibition of *THRA2*, which would clarify the precise mechanisms underlying this event, was not feasible owing to the minimal sequence divergence between *THRA1* and *THRA2*. No commercially validated siRNAs specifically targeting *THRA2* are currently available. Therefore, we designed and tested two independent *THRA2*-specific siRNAs (GACUGUGUCUGAAUCAUGU and GCUCCCCCAGGCAGAAAUA). However, neither achieved efficient or reproducible suppression of *THRA2* expression (data not shown). These technical constraints represent important limitations of the present study. Furthermore, *THRA1* and *THRA2* are generated from the same *THRA* locus through alternative splicing and are generally considered to share a common promoter region. To our knowledge, no isoform-specific promoter uniquely driving *THRA2* expression has been clearly established. Therefore, the identified demethylated region likely reflects regulatory changes at the *THRA* locus rather than a *THRA2*-specific promoter element. This structural constraint further limits the ability to attribute epigenetic changes exclusively to *THRA2*.

### Relationship between NCC-derived tumors and MEF2C or THRA2

*MEF2C* expression increased during differentiation into cNCCs in our system. Exploratory analysis of public datasets suggested that lower *MEF2C* expression may be associated with poorer prognosis in leiomyosarcoma (Figure S5). However, as this observation is based on external data and not directly tested in our experimental system, its clinical relevance remains speculative.

Our data suggest that *THRA2* undergoes DNA demethylation and increased expression during tNCC differentiation, whereas *THRA1* expression remains relatively unchanged, suggesting distinct regulatory mechanisms. Analysis of public neuroblastoma datasets indicated an association between low *THRA2* expression and poor prognosis (Figure S6). Given the presumed origin of neuroblastoma from tNCCs, this observation may be of interest; however, due to the lack of isoform-specific resolution in DNA methylation data and absence of direct functional validation, these findings should be interpreted with caution.

### Limitations of the study

This study has several limitations. DNA methylation microarray analyses were performed with a single measurement per cell line and condition (*n* = 1), without biological or technical replicates, limiting the assessment of variability. In addition, bulk transcriptomic and methylome analyses were used, which capture averaged signals across heterogeneous cell populations. This may obscure cell type-specific regulatory features and limit interpretation of lineage-specific conclusions. Although exploratory analysis of publicly available single-cell RNA sequencing datasets supported preferential expression patterns of *MEF2C* and *THRA*, these findings require further validation. Furthermore, time course and functional validation experiments were not performed, limiting insight into temporal dynamics and causal relationships. Therefore, these findings should be interpreted with caution and validated in independent studies using higher-resolution approaches.

## Resource availability

### Lead contact

Further information and requests for resources and reagents should be directed to and will be fulfilled by the lead contact, Kyosuke Mukae (kmukae@saitama-pho.jp).

### Materials availability

This study did not generate new unique reagents and material.

### Data and code availability

• Data: The gene expression and DNA methylation microarray data reported in this paper have been deposited in the GEO under accession numbers GSE286162 and GSE286164.
• Code: This paper does not report original code.
• Other items: Any additional information required to reanalyze the data reported in this paper is available from the lead contact upon request.

## Acknowledgments

We thank Editage (www.editage.jp) for English language editing. Financial support for this study was provided by the Saitama Cancer Center. This study was also supported by grants from the 10.13039/501100001691Japan Society for the Promotion of Science (10.13039/501100001691JSPS) 10.13039/501100001691KAKENHI (Grant no JP22K15517), Grant-in-Aid for Scientific Research (C) (Grant no JP24K10991), and Grant-in-Aid for Scientific Research (B) (Grant no 19H03625).

## Author contributions

K.M. and T.K. designed this study. K.M. performed most of the experiments. M.S., T.S., R.O., S.Y., N.H., T.U., and M.O. assisted in some experiments. K.M., M.O., and T.K. wrote the paper.

## Declaration of interests

The authors declare that they have no conflicts of interest.

## Declaration of generative AI and AI-assisted technologies in the writing process

During the preparation of this work, the authors used ChatGPT in order to improve language and readability. After using this tool or service, the authors reviewed and edited the content as needed and take full responsibility for the content of the publication.

## STAR★Methods

### Key resources table

**Table:** 

REAGENT or RESOURCE	SOURCE	IDENTIFIER
**Antibodies**		

Alexa Fluor® 647-conjugated mouse anti-human CD271 antibody	BD Biosciences	Cat#560326; RRID: AB_1645403
Anti-HOXC9 mouse monoclonal antibody	Santa Cruz Biotechnology	Cat#sc-81100; RRID: AB_2279855
Anti-PHOX2B rabbit polyclonal antibody	Proteintech	Cat#25276-1-AP; RRID: AB_2880001
Anti-PAX3 rabbit polyclonal antibody	Proteintech	Cat#21386-1-AP; RRID: AB_10732817
Anti-RPS18 rabbit polyclonal antibody	Aviva Systems Biology	Cat#ARP72659_P050
Anti-α-ACTIN polyclonal antibody	MilliporeSigma	Cat#A2066; RRID: AB_476693

**Chemicals, peptides, and recombinant proteins**		

primate ES cell medium	REPROCELL	Cat#RCHEMD001
recombinant human fibroblast growth factor 2	FUJIFILM	Cat#060-04543
Iscove’s modified Dulbecco’s medium	Thermo Fisher Scientific	Cat#12440053
Ham’s F-12	Thermo Fisher Scientific	Cat#11765054
chemically defined lipid concentrate	Thermo Fisher Scientific	Cat#11905031
apo-transferrin	Sigma-Aldrich	Cat#T1147
monothioglycerol	Sigma-Aldrich	Cat#M6145
BSA	Sigma-Aldrich	Cat#A8806
insulin	FUJIFILM	Cat#099-06473
penicillin/streptomycin	Thermo Fisher Scientific	Cat#15140122
fibronectin	MilliporeSigma	Cat#FC010
SB431542	Selleck	Cat#S1067
CHIR99021	FUJIFILM	Cat#034-23103
epidermal growth factor	R&D Systems	Cat#236-EG-200
retinoic acid	Sigma-Aldrich	Cat#R2625
BMP4	R&D Systems	Cat#314-BP-500/CF
proteinase K	FUJIFILM	Cat#166-28913
Nonidet P-40	NACALAI TESQUE, INC.	Cat#18551-24
random primer	TOYOBO	Cat#3801
reverse transcriptase	TOYOBO	Cat#TRT-101
rTaq	Takara Bio	Cat#R001A

**Critical commercial assays**		

ISOGENII	NIPPON GENE	Cat#311-07361
Lipofectamine RNAiMAX Transfection Reagent	Thermo Fisher Scientific	Cat#13778030
Clarity	Bio-Rad Laboratories	Cat#1705061
TB Green Premix DimerEraser	Takara Bio	Cat#RR091A

**Deposited data**		

Gene expression microarray	This study	GEO: GSE286162
DNA methylation microarray	This study	GEO: GSE286164
scRNA-seq (Zhao et al.)	Zhao et al., 2022	GEO: GSE168351
scRNA-seq (Xu et al.)	Xu et al., 2021	GEO: GSE157329

**Experimental models: Cell lines**		

Human: iPS cell line 414C2	Kyoto University	Provided by Kyoto University
Human: iPS cell line 201B7	Kyoto University	Provided by Kyoto University
Human: iPS cell line 604A3	Kyoto University	Provided by Kyoto University

**Oligonucleotides**		

Primers for PCR	This study	NA
siRNA Silencer Select Negative Control No. 1 siRNA	Thermo Fisher Scientific	Cat#4390843
siMEF2C	Thermo Fisher Scientific	Cat#s8653
siTHRA1/2	Thermo Fisher Scientific	Cat#s14116
Universal Negative Control siRNA	NIPPON GENE	NA
siTHRA2-1	NIPPON GENE	NA
siTHRA2-2	NIPPON GENE	NA

**Software and algorithms**		

GeneSpring GX	Agilent Technologies	https://www.chem-agilent.com/contents.php?id=1000215
BD FACSDiva Software	BD Biosciences	https://www.bdbiosciences.com/en-us
FlowJo	BD Biosciences	https://www.flowjo.com/
DAVID	Sherman et al.	https://david.ncifcrf.gov/
Enrichr	Ma’ayan Lab	https://maayanlab.cloud/Enrichr/
GenomeStudio Methylation Module Software	Illumina Inc.	https://support.illumina.com/downloads/genomestudio-2011-1-user-guides.html
MACON	Iida et al.	http://epigenome.ncc.go.jp/macon
R statistical software	R Core Team	https://www.r-project.org/
DMRcate v.4.5 R package	Bioconductor	https://bioconductor.org/packages/release/bioc/html/DMRcate.html
cpg.annotate v.1.8.6 R package	GitHub	https://www.rdocumentation.org/packages/DMRcate/versions/1.8.6/topics/cpg.annotate
dmrcate v.3.6.0 R package	Bioconductor	https://bioconductor.org/packages/release/bioc/html/DMRcate.html
findOverlaps v.2.6.1 R package	Bioconductor	https://www.rdocumentation.org/packages/IRanges/versions/2.6.1/topics/findOverlaps-methods
Seurat v.5.3.0 R package	Satija Lab	https://satijalab.org/seurat/
Matrix v.1.6-5 R package	CRAN	https://cran.r-project.org/web/packages/Matrix/index.html
ImageJ software 1.8.0	National Institute of Health	https://imagej.net/ij/
Prism9	GraphPad	https://www.graphpad.com/

**Other**		

8 × 60 K design Agilent platform	Agilent Technologies	Cat#G4851B
Infinium MethylationEPIC BeadChip v.1.0	Illumina Inc.	Cat#WG-317

### Experimental model and study participant details

#### Cell culture and differentiation methods

Human induced pluripotent stem cells (iPSCs) were used in this study. The 414C2 human iPSC line, derived from a female donor, was provided by Dr. Kenji Osafune (Kyoto University, Japan). The 201B7 and 604A3 human iPSC lines, derived from female and male donors, respectively, were provided by Dr. Junya Toguchida (Kyoto University, Japan). All cell lines were maintained on feeder layers of mitomycin C-treated embryonic mouse fibroblasts in primate ES cell medium (REPROCELL, Tokyo, Japan) supplemented with 4 ng/mL recombinant human fibroblast growth factor 2 (FUJIFILM, Tokyo, Japan). Differentiation of iPSCs into cNCCs or tNCCs was induced using previously described protocols.^15^^,^^16^^,^^17^ Cell line authentication was not performed. Mycoplasma contamination testing was not routinely performed at our institution. Sex-based differences were not specifically evaluated in this study.

The induction and maintenance of cNCCs were performed in chemically defined medium containing Iscove’s modified Dulbecco’s medium/Ham’s F-12 (1:1; Thermo Fisher Scientific, Waltham, MA, USA), 1× chemically defined lipid concentrate (Thermo Fisher Scientific), 15 mg/mL apo-transferrin (Sigma-Aldrich, St. Louis, MO, USA), 450 mM monothioglycerol (Sigma-Aldrich), 5 mg/mL purified BSA (Sigma-Aldrich), 7 mg/mL insulin (FUJIFILM, Tokyo, Japan), and penicillin/streptomycin (Thermo Fisher Scientific). Culture dishes were coated with fibronectin (MilliporeSigma, Burlington, MA, USA). cNCCs were induced using 10 μM SB431542 (Selleck, Osaka, Japan) and 1 μM CHIR99021 (FUJIFILM) and maintained using 20 ng/mL epidermal growth factor (R&D Systems, Minneapolis, MN, USA) and 20 ng/mL FGF2 (FUJIFILM). Following induction, NGFR-positive cNCCs were sorted using fluorescence-activated cell sorting (FACS) AriaIII (BD Biosciences, Franklin Lakes, NJ, USA) with an Alexa Fluor® 647-conjugated mouse anti-human CD271 (NGFR) antibody (560326; BD Biosciences), as previously described.^17^

The induction and maintenance of tNCCs were performed in Essential 6 medium (Thermo Fisher Scientific) on a 6-cm low-attachment dish (Sumitomo Bakelite, Tokyo, Japan). tNCCs were induced using 10 μM SB431542 and 2 μM CHIR99021 and maintained using 20 ng/mL FGF2, 100 nM retinoic acid (Sigma-Aldrich), and 50 ng/mL BMP4 (R&D Systems). The proportion of cells expressing PHOX2B, a tNCC marker, was analyzed using FACS Canto II (BD Biosciences) using an anti-PHOX2B (25276-1-AP; Proteintech, Rosemount, IL, USA). Differentiation into cNCCs and tNCCs was evaluated by assessing the gene expression of NCC markers using semiquantitative reverse transcription polymerase chain reaction (RT-PCR).

### Method details

#### Gene expression microarray

Total RNA was extracted from cells using ISOGENII (NIPPON GENE, Tokyo, Japan), and a microarray-based expression profiling was performed using an 8 × 60 K design ID G4851B Agilent platform (Agilent Single Color, 39494; Agilent Technologies, Santa Clara, CA, USA), according to the manufacturer’s instructions. Differentially expressed genes (DEGs) were identified by p-values lower than 0.05, using the moderated *t*-test after Benjamini–Hochberg corrections, and fold changes greater than 2 or lower than 0.5. Gene annotation was performed using GeneSpring GX (Agilent Technologies). DEGs annotated in GeneSpring GX were analyzed for the Kyoto Encyclopedia of Genes and Genomes (KEGG) and Gene Ontology (GO) using the Database for Annotation, Visualization, and Integrated Discovery (DAVID) Bioinformatics Resources v.6.8 (https://david.ncifcrf.gov/) online tool. Cell and transcription factor enrichment analysis was performed for the upregulated DEGs using Enrichr (https://maayanlab.cloud/Enrichr/).

#### DNA methylation analysis

Genomic DNA was isolated from iPSCs, cNCCs, and tNCCs derived from the 414C2, 201B7, and 604A3 cell lines using proteinase K digestion (FUJIFILM), followed by phenol-chloroform purification (NIPPON GENE). After RNase treatment and additional purification, DNA methylation analysis was conducted using the Infinium MethylationEPIC BeadChip v.1.0 (Illumina Inc., San Diego, CA, USA), as previously described.^38^^,^^39^ Briefly, the array was scanned with an iScan System (Illumina Inc.), and the data were processed using the GenomeStudio Methylation Module Software v2.0 (Illumina Inc.). Probe-level quality control was conducted using the MACON web tool.^40^ Probes with low-quality signals—defined by an intensity value of 0 or a detection p-value > 0.01—were excluded based on established criteria.^41^ Finally, 2,991 of the 865,918 probes were excluded. Subsequently, β-mixture quantile (BMIQ) normalization was conducted to adjust probe design biases. Each sample was analyzed only once owing to the high analytical reproducibility of this microarray.^42^ After filtering, 862,927 probes with β-values ranging from 0 (unmethylated) to 1 (fully methylated) were retained for analysis. To reduce the data size for convenient handling, neighboring probes (CpG sites) within 500 bp were assembled into genomic blocks using MACON. Probe annotation files for the Infinium MethylationEPIC BeadChip v1.0 were downloaded from the Illumina website (http://support.illumina.com/array/array_kits/infinium-methylationepic-beadchip-kit/downloads.html). Each genomic block was annotated based on its relative position to the transcription start site (TSS) and CpG island status. In total, 862,927 probes were assembled into 551,478 genomic blocks, of which 538,523 blocks located on autosomes were included in subsequent methylation analysis. The DNA methylation level of each genomic block was calculated as the mean β-value of all probes within that block. Among these blocks, 185,666 CpG islands were identified, with 57,248 corresponding to promoter regions, defined as TSS200, 5′ untranslated region (UTR), or first exon.

Differentially methylated regions (DMRs) were identified using the DMRcate package. CpG-level differential methylation was assessed using M-values and the limma linear modeling framework, with cell type (cNCC vs tNCC) specified in the design matrix and the contrast tNCC − cNCC applied. Significant CpGs were identified using the cpg.annotate function and aggregated into regions using DMRcate with default parameters. For each DMR, all constituent CpG sites were extracted, and effect sizes were calculated separately for each cell line (414C2, 201B7, and 604A3) as mean differences in M-values and β-values between tNCCs and cNCCs across CpGs within each DMR. CpG and gene annotations were obtained from the IlluminaHumanMethylationEPICanno.ilm10b4.hg19 package, and all CpG probe IDs and associated gene symbols overlapping each DMR were recorded. To assess functional relevance, overlap with distal enhancer-like signatures (dELS) was evaluated using ENCODE cCRE annotations, and genomic overlaps were determined with the findOverlaps function from the GenomicRanges package. For each DMR, enhancer overlap status and the number of overlapping dELS were recorded. DMRs were defined as significant if they showed an absolute methylation difference of at least 0.3 (|Δβ| > 0.3) and contained at least three CpG sites.

#### Small interfering RNA (siRNA) and transfection

Cells were transfected using 5 nM control siRNA (siRNA Silencer Select Negative Control No. 1 siRNA, 4390843; Thermo Fisher Scientific), MEF2C siRNA (s8653; Thermo Fisher Scientific), or thyroid hormone receptor alpha (THRA) (s14116; Thermo Fisher Scientific) using a Lipofectamine RNAiMAX Transfection Reagent (13778030; Thermo Fisher Scientific), according to the manufacturer’s instructions. The siRNA treatment was initiated at the onset of tNCC differentiation. For cNCCs, owing to cell damage caused by FACS, siRNA treatment was applied during the differentiation process after sufficient culture.

#### Semiquantitative RT-PCR and quantitative PCR (qPCR)

Total RNA (2 μg), prepared using ISOGENII, was reverse-transcribed to single-stranded cDNA using random primers (TOYOBO, Osaka, Japan) and reverse transcriptase (TOYOBO), according to the manufacturer’s instructions. PCR was performed using rTaq (R001A; Takara Bio, Shiga, Japan). qPCR was performed using a QuantStudio 7 Flex Real-Time PCR System (Applied Biosystems, Foster City, CA, USA), according to the manufacturer’s instructions with TB Green Premix DimerEraser (RR091A; Takara Bio). For normalization, *ACTIN* was used as a reference gene for tNCCs, whereas both *ACTIN* and *RPS18* were used for cNCCs. This strategy was based on established developmental lineage properties rather than observations specific to this study. cNCCs are known to generate mesenchymal derivatives, including muscle-related cell types, where cytoskeletal remodeling may influence *ACTIN* expression stability. Therefore, *RPS18* was included as an additional reference gene in cNCC analyses. In contrast, tNCCs predominantly give rise to neural lineages. Accordingly, they are less likely to undergo cytoskeletal changes that would compromise *ACTIN* stability. Based on this established biological understanding, *ACTIN* was considered an appropriate reference gene for tNCC normalization. qPCR experiments were performed with three technical replicates (n = 3). Statistical analyses (Student’s t-test) were conducted on these technical replicates to assess assay reproducibility. Therefore, the reported p-values reflect technical variability and should not be interpreted as evidence of biological significance. Primer sequences are presented in Table S16.

#### Western blot analysis

Cells were lysed in a buffer containing 0.5% Nonidet P-40 (NACALAI TESQUE, INC., Kyoto, Japan), 120 mM NaCl, 1 mM ethylenediaminetetraacetic acid, and 50 mM Tris–HCl (pH 7.5), supplemented with protease/phosphatase inhibitors.^43^ A total of 20 μg of protein per sample was separated *via* 10% sodium dodecyl sulfate-polyacrylamide gel electrophoresis to detect HOXC9 and PHOX2B expression. After electrophoresis, proteins were transferred to polyvinylidene difluoride membranes (MilliporeSigma). Membranes were blocked using Block ACE (KAC Co. Ltd., Kyoto, Japan) and incubated with an anti-HOXC9 mouse monoclonal antibody (sc-81100; Santa Cruz Biotechnology, TX, USA, 1:1000), anti-PHOX2B (Proteintech, 1:1000), anti-PAX3 rabbit polyclonal antibody (21386-1-AP; Proteintech, 1:1000), anti-RPS18 rabbit polyclonal antibody (ARP72659_P050; Aviva Systems Biology, San Diego, CA, USA), or anti-α-ACTIN polyclonal antibody (A2066; MilliporeSigma). The reacted proteins were detected using Clarity (1705061; Bio-Rad Laboratories, Hercules, CA, USA). Each series of experiments was conducted three times. Band intensities were quantified using ImageJ software 1.8.0 (National Institute of Health, Bethesda, MD, USA) and normalized to the band intensity of the internal control, ACTIN.

#### Single-cell RNA sequencing (RNA-seq) analysis

Publicly available single-cell RNA-seq datasets were used for comparative analysis of NCC populations. Mouse cNCCs were analyzed using the GSE168351 dataset, and human tNCCs were analyzed using the GSE157329 dataset. Raw count matrices and accompanying metadata were obtained from the Gene Expression Omnibus (GEO). Quality control was performed to remove low-quality cells based on standard criteria, including the number of detected genes and mitochondrial gene expression. Gene expression values were normalized and scaled using a standard log-normalization procedure. NCC populations were defined based on the expression of established marker genes. Mouse cNCCs were defined as cells exhibiting above-average expression of both *Sox10* and *Ets1*, whereas human tNCCs were defined as cells exhibiting above-average expression of both *SOX10* and *PHOX2B*. Cells showing above-average expression of *Sox10* or *SOX10* alone, but not the corresponding second marker, were classified as “Other NCC,” while cells with SOX10 expression at or below the average level were classified as “Non-NCC”. Single-cell–level expression of candidate genes was assessed within the defined NCC populations.

#### Ethics statement

This study was approved by the Ethics Committee of Saitama Cancer Center (approval number: 1655). All experiments involving human iPSCs were conducted in accordance with relevant guidelines and regulations. The human iPSC lines used in this study were generated from donor samples obtained with written informed consent in accordance with the Declaration of Helsinki and were provided by Kyoto University.

### Quantification and statistical analysis

Statistical analyses were performed using GraphPad Prism (GraphPad Inc., San Diego, CA, USA). Quantitative PCR (qPCR) data were analyzed using Student’s t-test (n = 3), and scRNA-seq data were analyzed using Dunnett’s multiple comparisons test. Statistical significance was set at *p* < 0.05.

## Supplemental information

Document S1. Figures S1–S6

Table S1. DAVID analysis (p < 0.01) of genes upregulated in cNCCs compared to iPSCs across all three iPSC–cNCC pairs

Table S2. DAVID analysis (p < 0.01) of genes upregulated in tNCCs compared to iPSCs across all three iPSC–tNCC pairs

Table S3. Cancer cell enrichment analysis (p < 0.05) of genes upregulated in cNCCs compared to iPSCs across all three iPSC–cNCC pairs, based on the Cancer Cell Line Encyclopedia

Table S4. Cancer cell enrichment analysis (p < 0.05) of genes upregulated in tNCCs compared to iPSCs across all three iPSC–tNCC pairs, based on the Cancer Cell Line Encyclopedia

Table S5. Transcription factor enrichment analysis (p < 0.05) of genes upregulated in cNCCs compared to iPSCs, consistently across all three iPSC–cNCC, using Enrichr Submissions TF-Gene Cooccurrence

Table S6. Transcription factor enrichment analysis (p < 0.05) of genes upregulated in tNCCs compared to iPSCs, consistently across all three iPSC–tNCC, using Enrichr Submissions TF-Gene Cooccurrence

Table S7. DNA methylation state of iPSCs, cNCCs, and tNCCs

Table S8. Genes that transitioned from a highly methylated state (β > 0.4) in iPSCs to a demethylated state (β < 0.1) in cNCCs

Table S9. DAVID analysis (p < 0.05) of genes listed in Table S8

Table S10. Genes that transitioned from a highly methylated state (β > 0.4) in iPSCs to a demethylated state (β < 0.1) in tNCCs

Table S11. DAVID analysis (p < 0.05) of genes listed in Table S10

Table S12. Pearson’s correlation coefficient analysis between DNA methylation (β-values of CpG sites) and gene expression

Table S13. DAVID analysis (p < 0.05) of genes that are both demethylated and upregulated in cNCCs compared to iPSCs across all three iPSC–cNCC pairs

Table S14. DAVID analysis (p < 0.05) of genes that are both demethylated and upregulated in tNCCs compared to iPSCs across all three iPSC–tNCC pairs

Table S15. Differentially methylated regions between tNCCs and cNCCs (tNCC vs. cNCC)

Table S16. List of primer sequences used for PCR
