# Supplementary material for: Epigenomic regulation of neural crest differentiation in human-induced pluripotent stem cells
Source: iScience. 2026 May 22;29(6):115993. doi: 10.1016/j.isci.2026.115993 (PMC13224019; doi:10.1016/j.isci.2026.115993)
Supplement: Document S1. Figures S1–S6 [file mmc1.pdf]

iScience, Volume *29*

Supplemental information

Epigenomic regulation

# of neural crest differentiation

in human-induced pluripotent stem cells

Kyosuke Mukae, Maya Shindo, Tianyuan Shi, Ritsuko Onuki, Satoshi Yamashita, Naoko Hattori, Toshikazu Ushijima, Miki Ohira, and Takehiko Kamijo

Figure S1

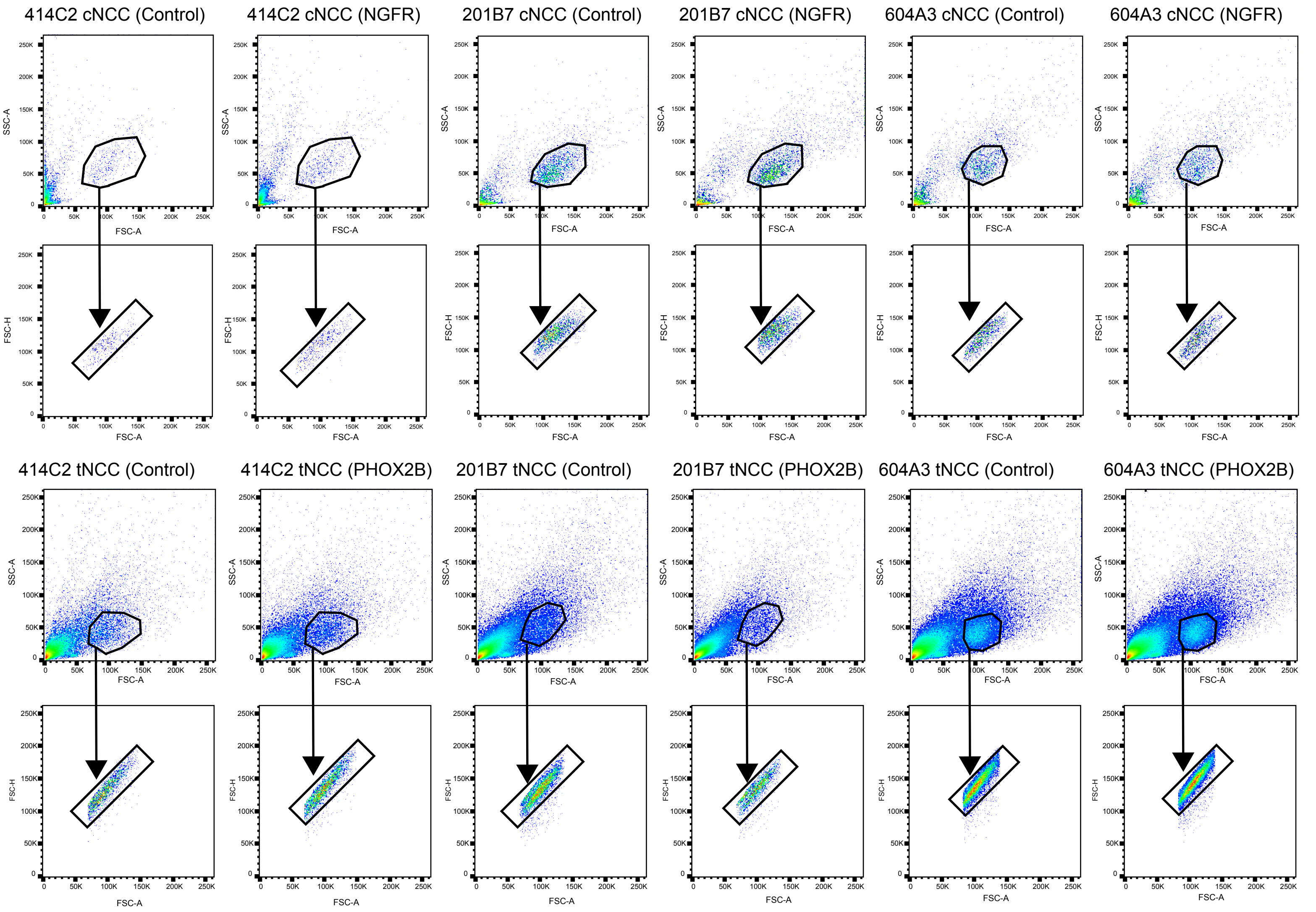

Figure S1. Flow cytometric gating strategy for identification of cNCC and tNCC populations derived from three independent iPSC lines (414C2, 201B7, and 604A3)

Representative flow cytometry plots showing the gating strategy used to define cNCCs and tNCCs. For each cell line, unstained controls and samples stained for NGFR- or PHOX2B are shown. Cells were first gated based on forward scatter (FSC-A) and side scatter (SSC-A) parameters to exclude debris. Subsequently, singlet discrimination was confirmed using FSC-A versus FSC-H. The final gated population of rectangle was used for downstream analysis and sorting. Comparable gating strategies were applied across all three iPSC lines. NGFR- or PHOX2B-positive populations were defined based on fluorescence intensity relative to the unstained control. Quantification of positive fractions is presented in Figure 1.

Figure S2

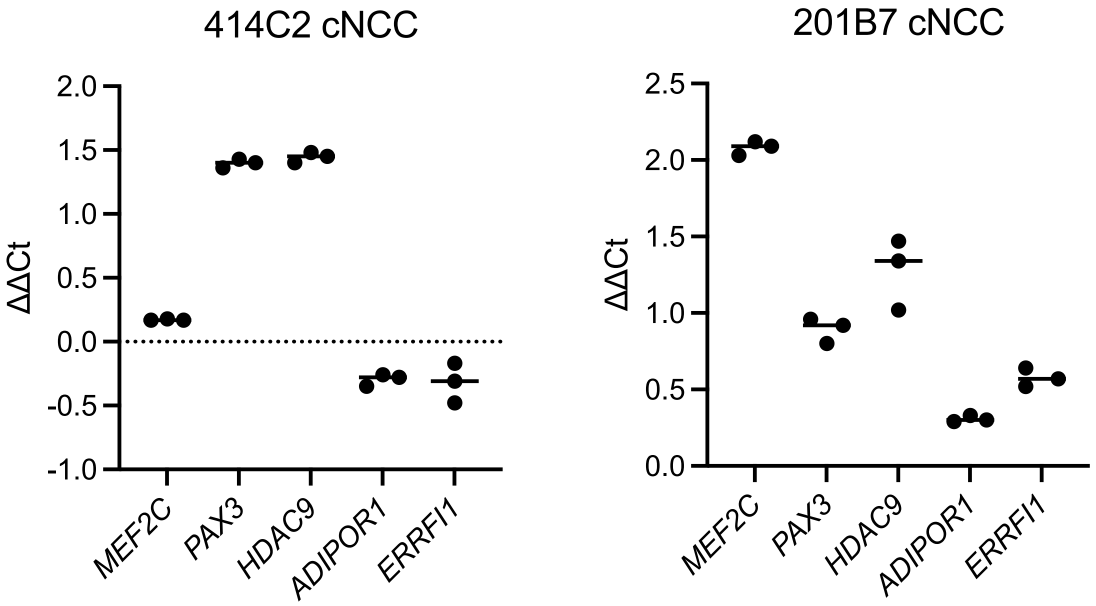

Figure S2. siRNA-mediated knockdown efficiency assessed by quantitative PCR (qPCR)

ΔΔCt-values were calculated as ΔCt(siRNA) − ΔCt(siControl), where ΔCt = Ct(target gene) − Ct(reference gene). *RPS18* was used as the reference gene for cNCCs, and *ACTIN* for tNCCs. Data are shown separately for each cell line (414C2 and 201B7). Each value represents the mean of three technical replicates derived from a single biological sample per cell line. Effect sizes are shown as ΔΔCt-values to assess cross-line reproducibility.

Figure S3

A

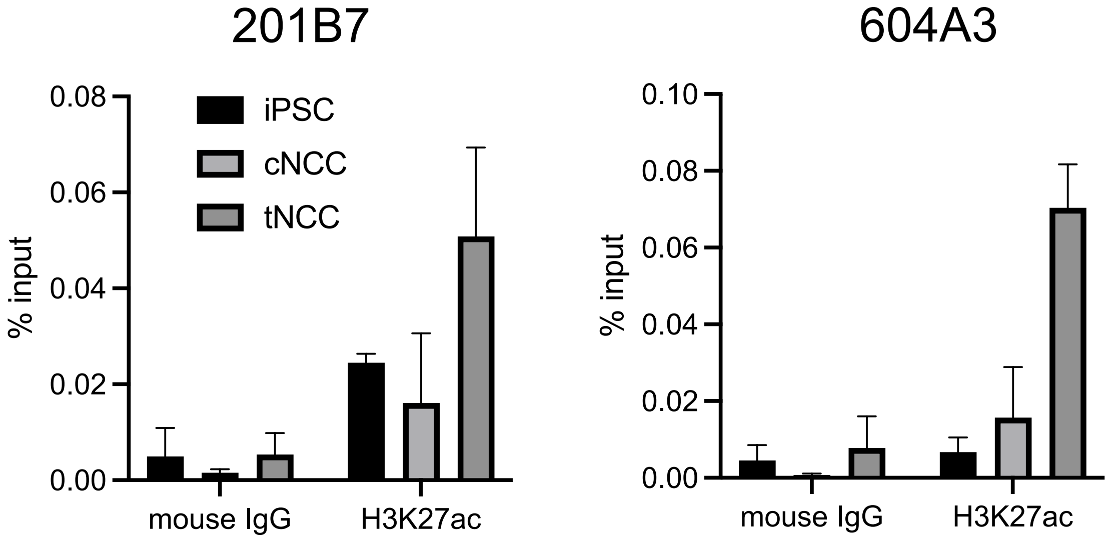

B

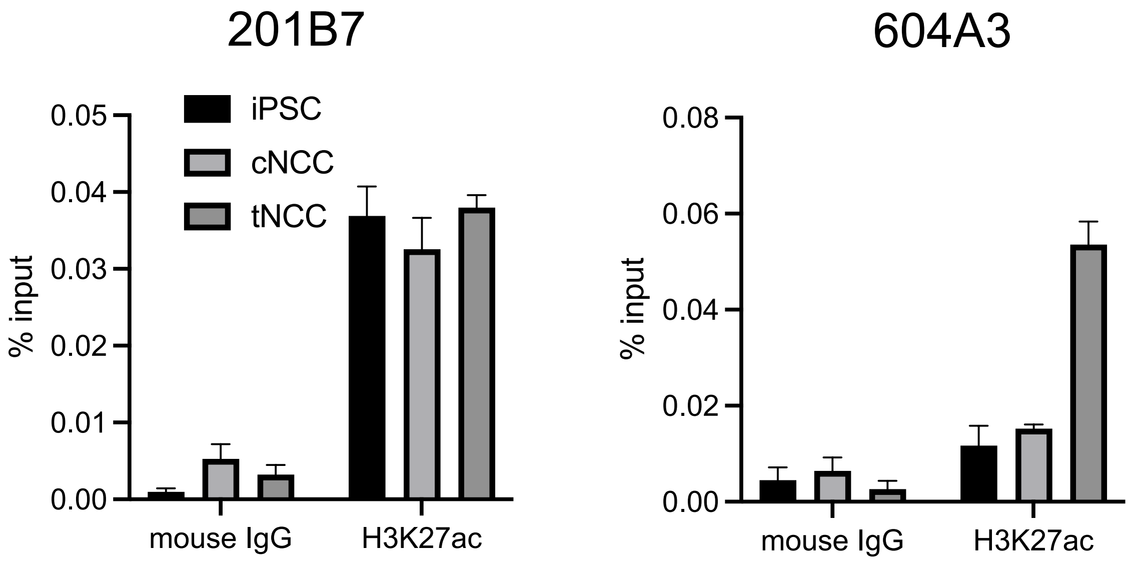

Figure S3. Promoter region of *MEF2C* and *THRA* locus

ChIP-qPCR was performed using iPSCs, cNCCs, and tNCCs derived from 201B7 and 604A3. The PCR region was set around the first exon of the *MEF2C* and *THRA* locus (Figures 6D and 7F). Immunoprecipitation was performed by an anti-H3K27ac antibody and control mouse IgG.

Figure S4

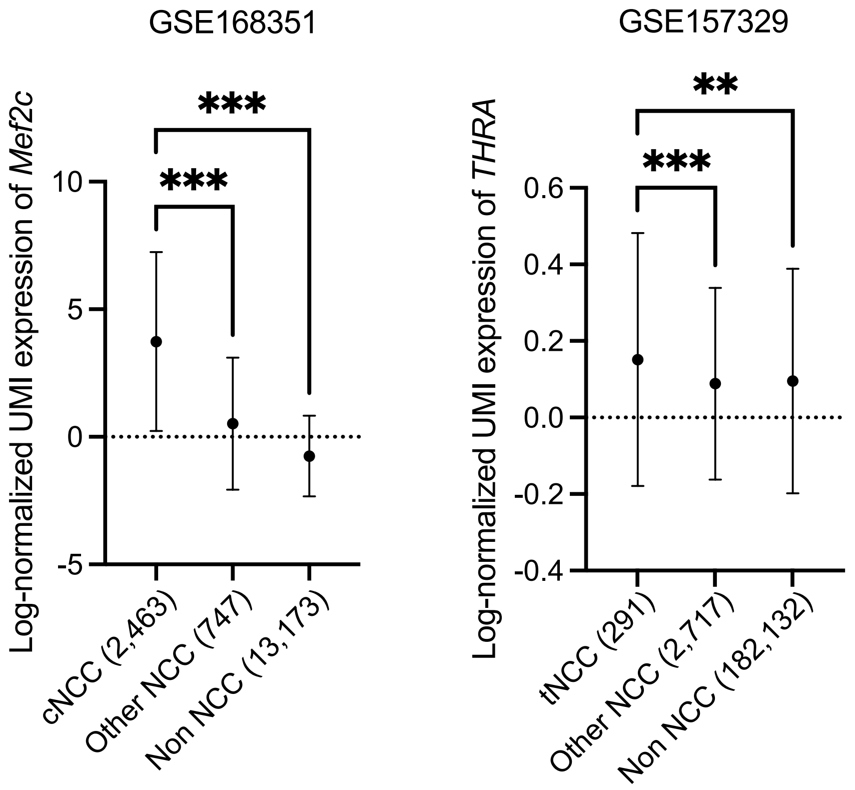

Figure S4. Exploratory analysis of publicly available single-cell RNA-seq datasets of neural crest cells

Mouse cNCCs were analyzed using dataset GSE168351, and human tNCCs using GSE157329. NCC populations were defined based on established marker genes. Mouse cNCCs were identified as cells exhibiting above-average expression of both *Sox10* and *Ets1*, whereas human tNCCs were defined as cells exhibiting above-average expression of both *SOX10* and *PHOX2B*. Cells expressing *Sox10* or *SOX10* above the average level but lacking concurrent expression of the corresponding second marker were classified as “Other NCC.” Cells with *Sox10*/*SOX10* expression at or below the average level were classified as “Non-NCC”. The numbers in parentheses indicate the sample size.

Figure S5

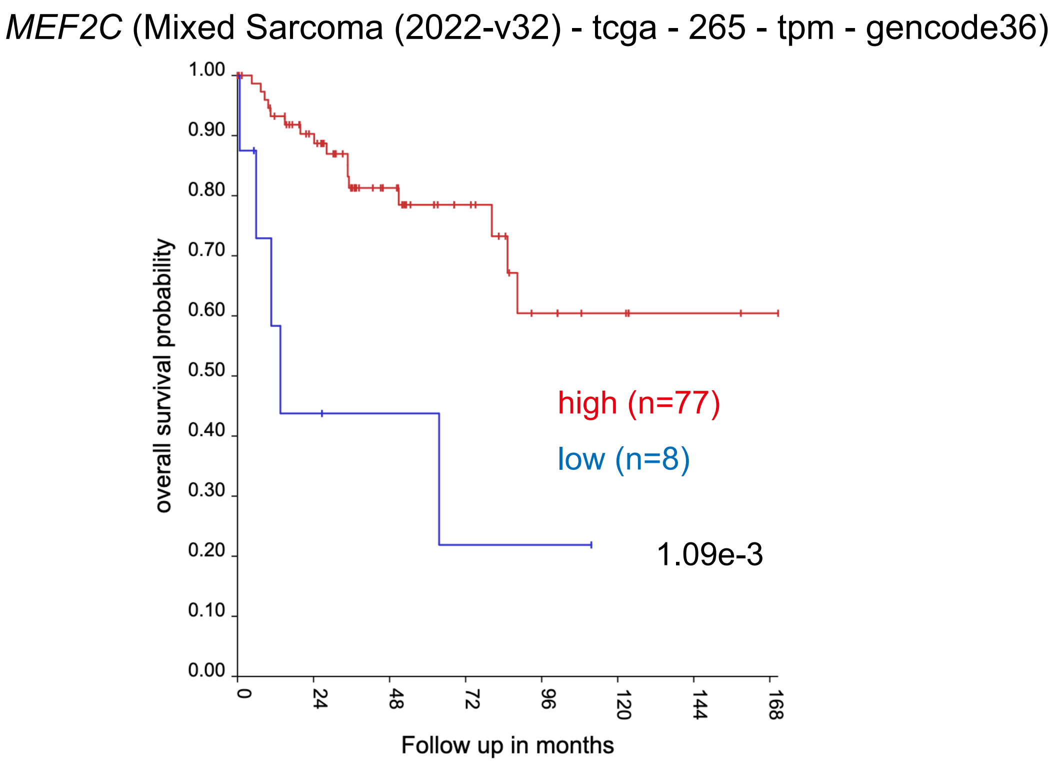

Figure S5. DNA methylation of *MEF2C* in smooth muscle and prognostic analysis of *MEF2C*

Kaplan–Meier analysis by R2 Genomics Analysis and Visualization Platform indicating overall survival related to *MEF2C* expression of in Mixed Sarcoma (2022-v32) - tcga - 265 - tpm - gencode36. Raw p-values are shown.

Figure S6

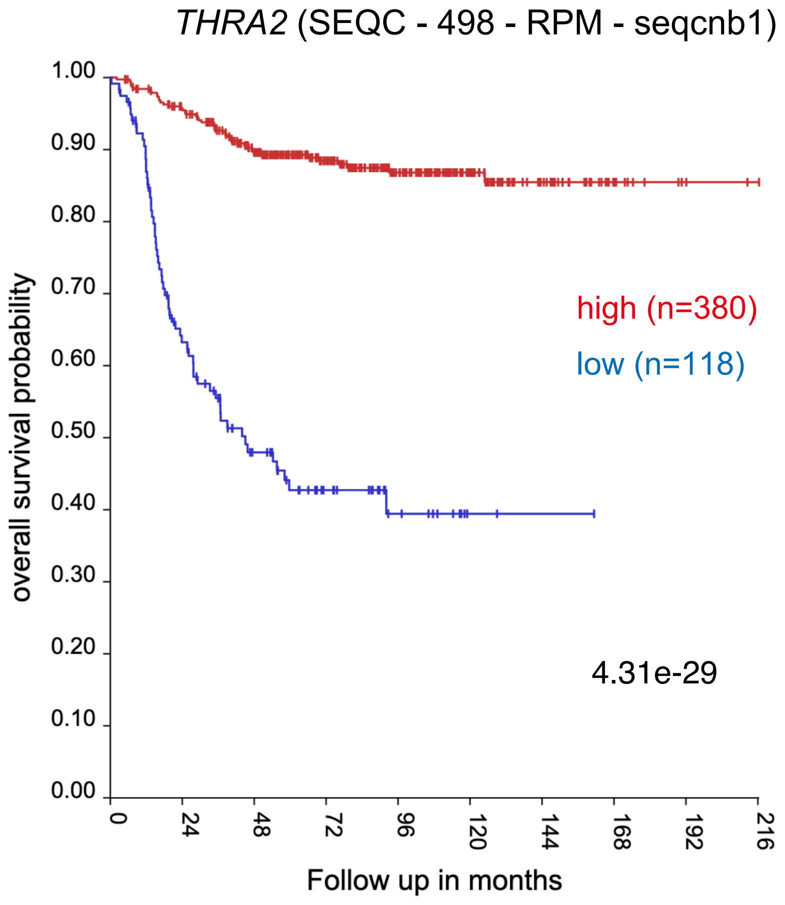

Figure S6. DNA methylation level of *THRA2* and prognostic analysis of *THRA2*

Kaplan–Meier analysis by R2 Genomics Analysis and Visualization Platform indicating overall survival related to *THRA2* expression in Tumor Neuroblastoma - SEQC - 498 - RPM - seqcnb1 (GSE62564). Raw p-values are shown.
